# Recombinant production of the antibody fragment D1.3 scFv with different *Bacillus* strains

**DOI:** 10.1186/s12934-017-0625-9

**Published:** 2017-01-23

**Authors:** Antonia Lakowitz, Rainer Krull, Rebekka Biedendieck

**Affiliations:** 1 Institute of Biochemical Engineering, Technische Universität Braunschweig, Rebenring 56, 38106 Braunschweig, Germany; 2 Center of Pharmaceutical Engineering (PVZ), Technische Universität Braunschweig, Franz-List-Straße 35a, 38106 Braunschweig, Germany; 3 Braunschweig Centre of Systems Biology (BRICS), Technische Universität Braunschweig, Rebenring 56, 38106 Braunschweig, Germany; 4 Institute of Microbiology, Technische Universität Braunschweig, Rebenring 56, 38106 Braunschweig, Germany

**Keywords:** *Bacillus megaterium*, *Bacillus licheniformis*, *Bacillus subtilis*, Antibody fragment D1.3 scFv, Recombinant, Secretion, Proteases, Scale-up cultivation, Scale-down cultivation

## Abstract

**Background:**

Different strains of the genus *Bacillus* are versatile candidates for the industrial production and secretion of heterologous proteins. They can be cultivated quite easily, show high growth rates and are usually non-pathogenic and free of endo- and exotoxins. They have the ability to secrete proteins with high efficiency into the growth medium, which allows cost-effective downstream purification processing. Some of the most interesting and challenging heterologous proteins are recombinant antibodies and antibody fragments. They are important and suitable tools in medical research for analytics, diagnostics and therapy. The smallest conventional antibody fragment with high-affinity binding to an antigen is the single-chain fragment variable (scFv). Here, different strains of the genus *Bacillus* were investigated using diverse cultivation systems for their suitability to produce and secret a recombinant scFv.

**Results:**

Extracellular production of lysozyme-specific scFv D1.3 was realized by constructing a plasmid with a xylose-inducible promoter optimized for *Bacillus megaterium* and the *D1.3scFv* gene fused to the coding sequence of the LipA signal peptide from *B. megaterium*. Functional scFv was successfully secreted with *B. megaterium* MS941, *Bacillus licheniformis* MW3 and the three *Bacillus subtilis* strains 168, DB431 and WB800N differing in the number of produced proteases. Starting with shake flasks (150 mL), the bioprocess was scaled down to microtiter plates (1250 µL) as well as scaled up to laboratory-scale bioreactors (2 L). The highest extracellular concentration of D1.3 scFv (130 mg L^−1^) and highest space–time-yield (8 mg L^−1^ h^−1^) were accomplished with *B. subtilis* WB800N, a strain deficient in eight proteases. These results were reproduced by the production and secretion of a recombinant penicillin G acylase (Pac).

**Conclusions:**

The genus *Bacillus* provides high potential microbial host systems for the secretion of challenging heterologous proteins like antibody fragments and large proteins at high titers. In this study, the highest extracellular concentration and space–time-yield of a recombinant antibody fragment for a Gram-positive bacterium so far was achieved. The successful interspecies use of the here-designed plasmid originally optimized for *B. megaterium* was demonstrated by two examples, an antibody fragment and a penicillin G acylase in up to five different *Bacillus* strains.

**Electronic supplementary material:**

The online version of this article (doi:10.1186/s12934-017-0625-9) contains supplementary material, which is available to authorized users.

## Background

Members of the genus *Bacillus* are rod-shaped aerobic or facultatively anaerobic, Gram-positive bacteria [1, 2]. This genus is one of the most diverse groups of microorganisms and its representatives are widely distributed in soil, air and water [1, 2]. Different *Bacillus* strains have been developed and engineered as industrial producers of natural enzymes such as alkaline proteases (*Bacillus clausii*), α-amylase (*Bacillus licheniformis*) and β-glucanase (*Bacillus subtilis*) [3], antibiotics like bacitracin (*B. licheniformis*) [4], insecticides such as δ-endotoxin (*Bacillus thuringiensis*) [5, 6], vitamin B_2_ (*B. subtilis*) [7], the supplement poly-γ-glutamic acid or ubiquinone biopolymer nanocarriers (*B. licheniformis*) [8, 9]. Besides natural products, *Bacillus* strains are great candidates for industrial production and secretion of heterologous proteins due to several advantages. In comparison to eukaryotic systems, their cultivation is simple and their high growth rates lead to short cultivation times. Most *Bacillus* strains are non-pathogenic and free of exo- and endotoxins. Species like *B. subtilis* and *B. licheniformis* even have the generally regarded as safe (GRAS) status [10, 11]. In addition, these species have the ability to secrete proteins directly into the extracellular medium, resulting in cost-effective downstream purification processing. In contrast, Gram-negative bacteria, like the best-analyzed representative *Escherichia coli*, accumulate proteins intracellularly and tend to build mostly cell-toxic and insoluble protein accumulations (inclusion bodies). These result in cost-intensive downstream processing due to incorrect protein folding and inefficient disulfide bridge formation [3, 12].

The production of proteins of eukaryotic origin in prokaryotic organisms provides one of the biggest challenges of biotechnology. Eukaryotic proteins heterologously produced and secreted with *Bacillus* are e.g. human interferon alpha (15 mg L^−1^, *B. subtilis*, [13]), growth hormone (200 mg L^−1^, *B. subtilis,* [14]) or epidermal growth factor (240 mg L^−1^, *Bacillus brevis*, [15]). Levels of these heterologous proteins are quite low in comparison to homologous proteins and those of prokaryotic (Gram-negative or -positive) origin, e.g. secreted α-amylase with up to 3 g L^−1^ [16]. One major limiting factor is the degradation of the secreted protein by membrane-bound, cell wall-associated or secreted proteases [17]. Proteases constitute the cellular quality control which degrade proteins folded too slowly or incorrectly [18]. Many heterologous proteins are frequently found to be inefficiently and slowly folded compared to the homologous counterparts since they are not coevolved with the host protein production machinery. This might even lead to the exposure of protease-recognition sequences even in correctly folded conformation [16, 19, 20]. To overcome degradation of secreted heterologous proteins, many protease-deficient *Bacillus* mutants have been successfully constructed and used resulting in more effective extracellular protein production [21].

Some of the most interesting and challenging proteins heterologously produced in prokaryotes are recombinant antibodies and antibody fragments which are important and suitable tools in research and medicine. Their specific antigen binding is used in analytics, in proteome research, in diagnostics of pathogens and toxins and in therapy of inflammatory and tumor diseases [22–24]. A whole immunoglobulin G molecule is a hetero-tetramer with two heavy and two light chains connected by disulfide bridges and intramolecular disulfide bridges for stabilization [25]. Since their production requires a complicated folding apparatus and an oxidizing environment for disulfide bridge formation, many microbial host systems fail to produce significant amounts of the molecules [26]. In addition, bacterial hosts usually do not accomplish the correct glycosylation of the produced antibodies. However, smaller and simpler antibody fragments with full antigen binding capacity have been developed for research purposes, where biological activity is more important than structural authenticity and correct glycosylation pattern. The smallest conventional antibody fragment with high-affinity binding to an antigen is the single-chain fragment variable (scFv) [27], a heterodimer comprising the antibody heavy- and light-chain variable domains connected by a peptide linker and with natural disulfide bonds within the chains to stabilize the molecule [28].

Here, the recombinant production and secretion of the lysozyme-specific antibody fragment D1.3 scFv in *B. megaterium*, *B. licheniformis* and three *B. subtilis* strains differing in their protease equipment are presented to demonstrate the interspecies use of the *B. megaterium* plasmid system. For this, the gene encoding His-tagged D1.3 scFv genetically fused to a suitable secretion signal was expressed under the control of a xylose-inducible promoter optimized for recombinant protein production in *B. megaterium*. Starting with uncontrolled shake flask (150 mL culture volume) cultivations, the bioprocess was scaled down to microtiter plates (1250 µL culture volume), but also scaled up to controlled laboratory-scale stirred tank bioreactors (2 L culture volume). Growth, secretion behavior, product titer, product kinetics and space–time-yield (STY) were compared between the different strains and cultivation systems. Finally, these results were reproduced for the production and the secretion of a recombinant penicillin G acylase (Pac) with a three times higher molecular weight compared to the D1.3 scFv. Pac (EC 3.5.1.11) catalyzes the cleavage of penicillin G and cephalosporin G to intermediates important for the production of semi-synthetic β-lactam antibiotics [29]. It is autocatalytically processed from a single cytoplasmic polypeptide precursor carrying a signal peptide and a spacer peptide and finally correctly folded outside the cell before released to the growth medium [30, 31]. Therefore, Pac is a challenging candidate for recombinant secretion carried out here with three different *Bacillus* strains using the same plasmid background as for the recombinant D1.3 scFv.

## Results

### Plasmid for the recombinant production and secretion of D1.3 scFv

**Fig. 1:**
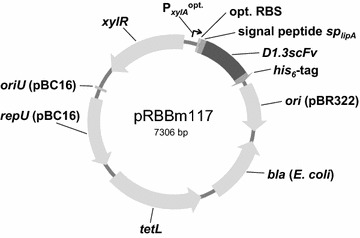
Plasmid pRBBm117 for recombinant production of the antibody fragment D1.3 scFv. P_*xylA*_^opt.^, optimized xylose-inducible promoter; opt. RBS, optimized ribosome binding site; *sp*_*lipA*_, signal peptide sequence of *B. megaterium* extracellular esterase LipA; *D1.3scFv*, gene for single chain fragment variable (scFv) against lysozyme; *his*_*6*_-tag, synthetic tag for 6× histidine residues; *bla*, β-lactamase gene for ampicillin resistance; *ori* (pBR322), *E. coli* origin of replication from plasmid pBR322; *oriU* (pBC16), *B. megaterium* origin of replication from plasmid pBC16; *repU* (pBC16), gene essential for plasmid replication in *B. megaterium* from plasmid pBC16; *tetL*, gene encoding tetracycline efflux pump for tetracycline resistance; *xylR*, gene encoding xylose repressor

The xylose-inducible promoter P_*xylA*_ was originally derived from the *B. megaterium* strain DSM319. There it controls the *xylABT* operon encoding proteins of xylose uptake and metabolism via a divergently encoded repressor protein, termed XylR. P_*xylA*_ was systematically optimized for high yield protein production in *B. megaterium* previously [32]. For the production, secretion and purification of D1.3 scFv in *B. megaterium*, the corresponding synthetic gene was fused to the coding sequences for the signal peptide of the lipase A of *B. megaterium* and for a his_6_-tag under control of native P_*xylA*_ before [33]. The employed vector backbone was carrying the *oriU*, *repU* and *tetL* genes from the *B. cereus* plasmid pBC16 [34] for replication and selection in *Bacillus*. To now enhance the recombinant production and secretion of D1.3 scFv, the *D1.3scFv* gene fused to the lipase A signal peptide coding sequence was cloned into a pBC16-derived plasmid carrying the P_*xylA*_, optimized for recombinant protein production in *B. megaterium* (Fig. 1). Furthermore, this new plasmid pRBBm117 is minimized by around 700 bp to reduce metabolic burden for the production strain [35].

**Fig. 2:**
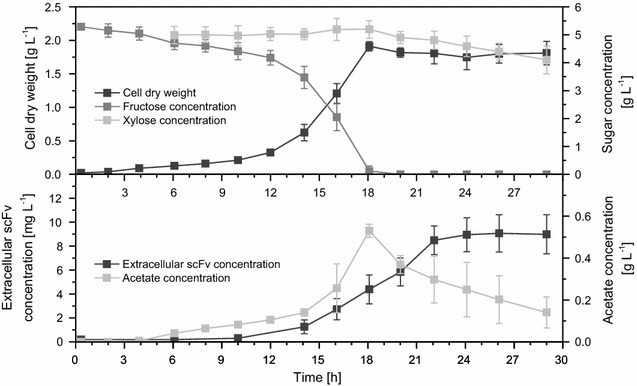
Cultivation profile of recombinant *B. megaterium* MS941 secreting D1.3 scFv in shake flasks. *B. megaterium* MS941 was transformed with plasmid pRBBm117 (P_*xylA*_^opt.^-*sp*_*lipA*_-*d1.3scFv*-*his*_*6*_). Cultivation took place in baffled shake flasks (150 mL culture volume) under aerobic conditions at 37 **°**C. Cultures were inoculated with cell concentrations (cell dry weight) of 0.0223 g L^−1^ in minimal medium containing 10 mg L^−1^ of tetracycline. Recombinant D1.3 scFv secretion was induced by the addition of 5 g L^−1^ of xylose at cell concentrations of around 0.07 g L^−1^. Samples were taken at given time points to analyze cell dry weight [g L^−1^], extracellular concentrations of D1.3 scFv [mg L^−1^] using ELISA and of fructose, xylose and acetate using HPLC

After transformation of *B. megaterium* MS941, a mutant of DSM319 lacking the main extracellular protease NprM and thus around 98% of extracellular protease activity [36], the recombinant secretion of D1.3 scFv was analyzed in 150 mL shake flask cultivation in minimal medium (Table 1). The oxygen input was realized via orbital shaking movements. The volumetric mass transfer coefficients for oxygen (*k*_*L*_ *a*) of 30–50 h^−1^ were assumed [37, 38]. Growth passed the typical phases of a batch process (Fig. 2). The addition of 5 g L^−1^ xylose for the induction of recombinant gene expression 2–3 h after inoculation led to temporarily decreased growth. The specific growth rate µ [h^−1^] is the proportionality factor between time-dependent change of cell concentration dX/dt [g L^−1^ h^−1^] and current cell concentration X [g L^−1^] (dX/dt = µ · X). It decreased from 0.45 to 0.15 h^−1^. Growth became accelerated again (0.33 h^−1^) until the initial 5 g L^−1^ fructose were completely consumed after 16–18 h. Stationary phase was attained with about 2 g L^−1^ of cell dry weight. The secretion of active, correctly folded D1.3 scFv already started during the exponential growth phase while maximal amounts of 8 mg L^−1^ were detected 4 h after entering the stationary phase and stayed constant until the end of the cultivation. The production was assumed mixed-growth-associated: the growth-associated biomass-related production yield coefficient α ≡ Y_P/X_ = 0.13 ${\text{mg}}_{\text{scFv}} \,{\text{g}}_{{_{\text{cells}} }}^{ - 1}$ and the non-growth-associated specific production rate β = 0.41 ${\text{mg}}_{\text{scFv}} \,{\text{g}}_{{_{\text{cells}} }}^{ - 1} \,{\text{h}}^{{{ - }1}}$ were calculated (Additional file 1: Table S1). During stationary phase, the carbon source fructose was already depleted and the formerly secreted acetate was consumed. The concentration of the inducer xylose stayed constant at a level of 5 g L^−1^ until fructose was exhausted and was only slightly reduced to >4 g L^−1^ until the end of the cultivation (Additional file 1: Figure S1). Little consumption of xylose by *B. megaterium* MS941 after depletion of the main carbon source was described earlier [30] and only slightly influences the amount of recombinant protein [39]. Due to the used optimized xylose-inducible promoter P_*xylA*_ the D1.3 scFv concentration was found to be around tenfold enhanced compared to data published by David et al. [40].

**Table 1:** Summary of cultivation conditions in microtiter plates, shake flasks and bioreactors

Parameter	Microtiter plate	Shake flask	Bioreactor
Cultivation volume [mL]	1.25	150	2000
Shake/stirrer frequency [min^−1^]	1000^a^	130^b^	500
Aeration rate [L min^−1^] with air	–	–	3
*k*_*L*_ *a* value [h^−1^]	150–200	30–50	75–85^c^
pH value [–]	Unregulated	Unregulated	6.5

^a^3 mm orbital diameter

^b^50 mm orbital diameter

^c^Before addition of anti-foaming agent

### Bioprocess scale-down and scale-up

**Fig. 3:**
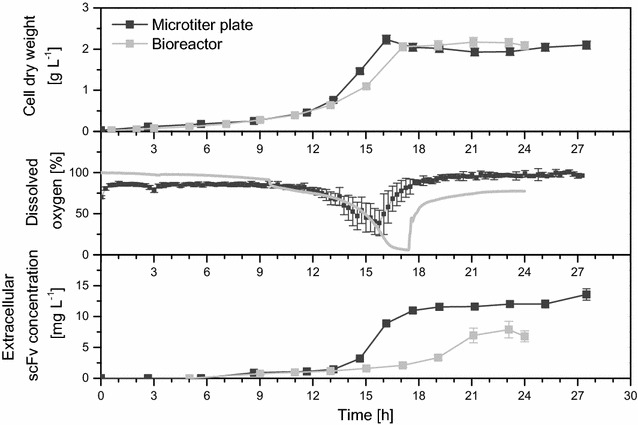
Cultivation profile of recombinant *B. megaterium* MS941 secreting D1.3 scFv in microtiter plates and bioreactors. *B.* *megaterium* MS941 was transformed with plasmid pRBBm117 (P_*xylA*_^opt.^-*sp*_*lipA*_-*d1.3scFv*-*his*_*6*_). Cultivation took place in microtiter plates (1250 μL culture volume) and stirred tank bioreactors (2 L culture volume) under aerobic conditions at 37 **°**C. All cultivation systems were inoculated with cell concentrations (cell dry weight) of 0.0223 g L^−1^ in minimal medium containing 10 mg L^−1^ of tetracycline. Recombinant D1.3 scFv secretion was induced by the addition of 5 g L^−1^ of xylose at cell concentrations of around 0.07 g L^−1^. Samples were taken at given time points to analyze cell dry weight [g L^−1^] and extracellular concentration of D1.3 scFv [mg L^−1^] using ELISA. Dissolved oxygen was measured online. Further analyses are shown in Additional file 1: Figure S1

Next, the culture volume was either reduced to 1250 μL or increased to 2 L (Table 1). While miniaturization from mL to µL scale using microtiter plates is advantageous for processing a large number of parallel experiments, lab-scale stirred tank bioreactors provide more opportunities for online measuring and controlling various cultivation parameters. One of the important parameters in this study was the oxygen supply. For shake flasks and microtiter plates, oxygen input was realized via orbital shaking movements. As the ratio of gas–liquid exchange area to liquid volume strongly increased in microtiter plates compared to that provided in the shake flasks [41], a higher oxygen transfer rate (OTR) with *k*_*L*_ *a* values of 150–200 h^−1^ was obtained. For stirred tank bioreactors, oxygen input was ensured via direct air inflow and agitation and *k*_*L*_ *a* values of 75–85 h^−1^ were measured. However, addition of 15 mg L^−1^ anti-foaming agent after around 9 h of cultivation was necessary in bioreactor cultivations. Anti-foaming agents usually decreases oxygen transfer due to coalescence of bubbles and by retarding surface flow at the gas liquid interface [42, 43]. The DO dropped to nearly 0% and a sudden rise of dissolved oxygen (DO) to 80% marked the transition to the stationary phase in the bioreactor cultivations (Fig. 3). In the microtiter plates, the DO declined to only 40% due to better oxygen transfer. Lower oxygen tension slowed the energy generation of the respiratory chain and the present fructose concentration caused overflow metabolism [44], indicated by the secretion of acetate during growth (Fig. 2 and Additional file 1: Figure S1) which has been described previously [40, 45]. In this study, maximal acetate concentrations of 0.45–0.55 g L^−1^ were attained in shake flask and bioreactor cultivations, but only 0.25 g L^−1^ in microtiter plate experiments. Acetate as non-preferential substrate was re-utilized after fructose depletion in shake flasks and microtiter plates. Finally, the oxygen availability also influences the amount of recombinant D1.3 scFv produced (Fig. 3). While in the bioreactor the production profile of D1.3 scFv was found to be very similar to that in shake flasks with maximal concentrations of 8 mg L^−1^ after 22–23 h of cultivation, in microtiter plates higher concentrations of up to 15 mg L^−1^ were reached already after 17–18 h. As the product was secreted in both growth and stationary phase, secretion was assumed to be mixed-growth-associated or slightly growth-independent with α = 0.28 ${\text{mg}}_{\text{scFv}} \,{\text{g}}_{{_{\text{cells}} }}^{ - 1}$ and β = 0.79 ${\text{mg}}_{\text{scFv}} \,{\text{g}}_{{_{\text{cells}} }}^{ - 1} \,{\text{h}}^{{{ - }1}}$ for microtiter plate cultivations and α = 0 ${\text{mg}}_{\text{scFv}} \,{\text{g}}_{{_{\text{cells}} }}^{ - 1}$ and β = 0.31 ${\text{mg}}_{\text{scFv}} \,{\text{g}}_{{_{\text{cells}} }}^{ - 1} \,{\text{h}}^{{{ - }1}}$ for bioreactor experiments (Additional file 1: Table S1). The amount of active antibody fragment remained constant also in late stationary phase or even further increased during microtiter plate cultivation (Figs. 2, 3) indicating a stable product form.

### Production of recombinant D1.3 scFv using *B. licheniformis*

Xylose-inducible gene expression can also be found in *B. licheniformis* [46] indicating its ability to take up xylose present in the medium. Hence, the plasmid pRBBm117 constructed for recombinant xylose-dependent D1.3 scFv secretion in *B. megaterium* was introduced into *B. licheniformis* strain MW3 [47]. Strain MW3 is a restriction-negative mutant of the type strain DSM13 in which two type I restriction modification systems were destructed enabling a better transformation and higher stability of foreign DNA compared to the wild type [47].

**Fig. 4:**
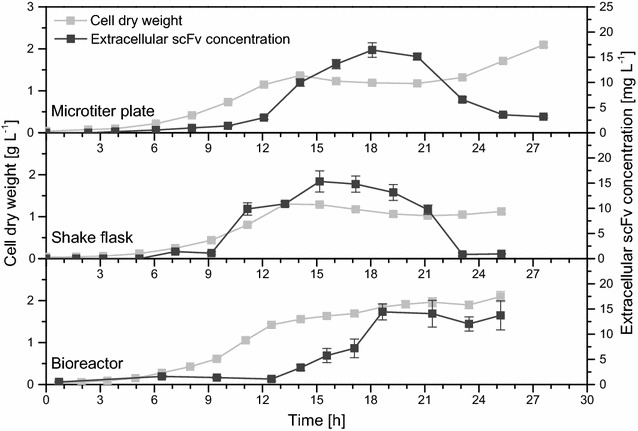
Cultivation profile of recombinant *B. licheniformis* MW3 secreting D1.3 scFv. *B.* *licheniformis* MW3 was transformed with plasmid pRBBm117 (P_*xylA*_^opt.^-*sp*_*lipA*_-*d1.3scFv*-*his*_*6*_). Cultivation took place in microtiter plates (1250 μL culture volume), baffled shake flasks (150 mL of culture volume) and stirred tank bioreactors (2 L of culture volume) under aerobic conditions at 37 °C. All cultivation systems were inoculated with cell concentrations (cell dry weight) of 0.04 g L^−1^ in minimal medium containing 10 mg L^−1^ of tetracycline. Recombinant D1.3 scFv secretion was induced by the addition of 5 g L^−1^ of xylose at cell concentrations of around 0.12 g L^−1^. Samples were taken at given time points to analyze cell dry weight [g L^−1^] and extracellular concentrations of D1.3 scFv [mg L^−1^] using ELISA. Further analyses are shown in Additional file 1: Figure S2

The growth behavior of recombinant *B. licheniformis* MW3 (Fig. 4) was similar for all three cultivation systems with maximal specific growth rates of 0.34–0.42 h^−1^. In contrast to recombinant *B. megaterium*, growth was not notably reduced after addition of the inducer xylose 4–5 h after inoculation. In all three systems, fructose was consumed after 12–14 h and stationary phase was reached with about 1.5–2.0 g L^−1^ of cell dry weight. Less maximal biomass was formed compared to recombinant *B. megaterium* MS941.

In the bioreactor cultivation, DO decreased to nearly 0% at the end of the exponential growth phase (Additional file 1: Figure S2) implying oxygen limitation although fructose was still available. In microtiter plates, better oxygen transfer resulted in a less strongly decrease of DO to 50–60%. As for recombinant *B. megaterium,* this was accompanied by the secretion of the overflow metabolite acetate. Maximal acetate concentrations of 0.7 g L^−1^ were detected in bioreactors, but only 0.4 g L^−1^ in shake flaks and microtiter plates when the culture reached the stationary phase. Re-utilization of the secreted acetate as well as partly metabolization of xylose explains the slight biomass increase after fructose depletion in bioreactors and microtiter plates. Secretion and re-utilization of acetate [48] and the utilization of xylose as carbon source [49] were described previously.

Although growth was not affected by the addition of the inducer, slightly higher amounts of recombinant functional D1.3 scFv were secreted by recombinant *B. licheniformis* compared to *B.* *megaterium*. Highest product amounts were again reached in microtiter plates (17 mg L^−1^ after 18 h), followed by shake flasks (15 mg L^−1^ after 15 h) and bioreactor cultivations (13 mg L^−1^ after 18 h). Secretion seems to be non-growth-associated in microtiter plates (α = 0 ${\text{mg}}_{\text{scFv}} \,{\text{g}}_{{_{\text{cells}} }}^{ - 1}$ and β = 1.19 ${\text{mg}}_{\text{scFv}} \,{\text{g}}_{{_{\text{cells}} }}^{ - 1} \,{\text{h}}^{{{ - }1}}$) and bioreactors (α = 0 ${\text{mg}}_{\text{scFv}} \,{\text{g}}_{{_{\text{cells}} }}^{ - 1}$ and β = 0.51 ${\text{mg}}_{\text{scFv}} \,{\text{g}}_{{_{\text{cells}} }}^{ - 1} \,{\text{h}}^{{{ - }1}}$) as occurring mainly in the stationary phase (Additional file 1: Table S1). For shake flask cultivation, mixed-growth-associated secretion was observed with α = 3.286 ${\text{mg}}_{\text{scFv}} \,{\text{g}}_{{_{\text{cells}} }}^{ - 1}$ and β = 1.286 ${\text{mg}}_{\text{scFv}} \,{\text{g}}_{{_{\text{cells}} }}^{ - 1} \,{\text{h}}^{{{ - }1}}$ (Additional file 1: Table S1). For cultivations in microtiter plates and shake flasks, extracellular scFv concentration decreases during stationary phase. Since *B. licheniformis* DSM13, the wild type strain of the here-used derivate MW3, is known to produce various extracellular proteases including well-studied Subtilisin Carlsberg [50], degradation of scFv by these enzymes is assumed.

### Influence of genome-encoded proteases on the amount of secreted D1.3 scFv in recombinant *B. subtilis*

Next, three different *B. subtilis* strains, 168 [51], DB431 [52] and WB800N [53, 54], were individually transformed with the production plasmid pRBBm117. The three *B. subtilis* strains differ in their gene sets for intra- and extracellular proteases what should strongly influence the stability of recombinant D1.3 scFv inside and outside of the cell. Compared to the wild type strain *B. subtilis* 168, *B. subtilis* strain DB431 is deficient in four extracellular [subtilisin E (AprE), neural metalloprotease (Npr), minor extracellular protease (Epr), extracellular metalloprotease (Mpr)] and two intracellular proteases [the major intracellular serine protease (IspA) and bacillopeptidase F (Bpr)] [52, 55]. *B. subtilis* strain WB800N is also a derivative of strain 168 and lacks six extracellular proteases namely subtilisin E (AprE), neural protease B (NprB), bacillolysin (NprE), two minor extracellular proteases (Epr and Vpr) and metalloprotease (Mpr) as well as a cell wall-associated protease (WprA) and intracellular bacillopeptidase F (Bpr) [53–55].

**Fig. 5:**
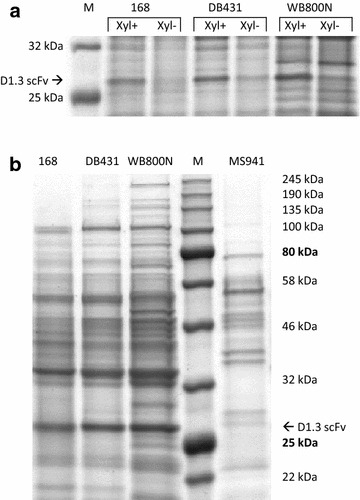
Secretion of D1.3 scFv and other extracellular proteins by recombinant *Bacillus* strains. Extracellular proteins of 188 µL culture supernatant from shake flasks were precipitated and analyzed by SDS-PAGE. **a** Extracellular proteins of *B. subtilis* strains 168, DB431 and WB800N were analyzed by SDS-PAGE after 13–15 h of cultivation. Xylose-induced (Xyl+) and non-induced (Xyl−) cultures were compared with a protein standard (*marker M*). Only the proteins with a size between 25 and 32 kDa are shown. **b** Extracellular proteins of induced *B. megaterium* MS941 (22 h) and *B. subtilis* 168, DB431 and WB800N (13–15 h) were precipitated and analyzed by SDS-PAGE compared with a protein standard (*marker M*)

Since *B. subtilis* was described to ineffectively utilize xylose as carbon source due to a missing specific transport system [56], the suitability of the here used and optimized xylose-inducible plasmid system had to be tested for the used strains. For this purpose, extracellular precipitated proteins were compared after xylose-induced and non-induced cultivations after 13–15 h in shake flasks via SDS-PAGE. In the medium of all three *B. subtilis* strains the antibody fragment D1.3 scFv, a monomeric protein with a molecular weight of approximately 27 kDa, was clearly observed for induced, but only marginally for non-induced cultivations (Fig. 5a). This indicated the enhanced production and secretion of D1.3 scFv in the presence of xylose and confirmed the correct function of the here used xylose-inducible promoter system. At the same time, the used plasmid system revealed a high segregational stability in the here tested *B. subtilis* strains. The application of the xylose-inducible promoter in *B. subtilis* was described previously but was only tested as genomically integrated system due to plasmid instabilities [57]. In addition, the amount of secreted D1.3 scFv positively correlated with the number of eliminated proteases. *B. subtilis* WB800N showed the highest and *B. subtilis* 168 the lowest concentration of D1.3 scFv (Fig. 5b). Although *B. megaterium* strain MS941 showed a very low protease activity [36], there was only very low extracellular overall protein and corresponding very low amount of extracellular D1.3 scFv visible compared to WB800N (Fig. 5b).

### Production and secretion of recombinant penicillin G acylase in different *Bacillus* strains

To verify obtained results for the recombinant production of D1.3 scFv similar experiments were performed for the recombinant production of the exo-enzyme penicillin G acylase (Pac) using *B. megaterium* MS941, *B. subtilis* 168 and *B. subtilis* WB800N. Pac consists of a small α subunit (27 kDa) and a large β subunit (59 kDa) which are autocatalytically processed from a cytoplasmic polypeptide precursor and folded outside the cell after secretion [30, 31]. For recombinant production of Pac in different *Bacillus* strains, the expression plasmid pALBm1 was constructed, which encodes the Pac protein from *B. megaterium* ATCC14945 with its original signal peptide. Transcription of the corresponding gene is controlled by the optimized xylose-inducible promoter system. All other structures of pALBm1 are identical to these of the plasmid pRBBm117 used for antibody fragment production. Cultivations of transformed *B. megaterium* MS941 as well as *B. subtilis* 168 and WB800N were performed in shake flasks with 150 mL of LB medium at 37 °C.

**Fig. 6:**
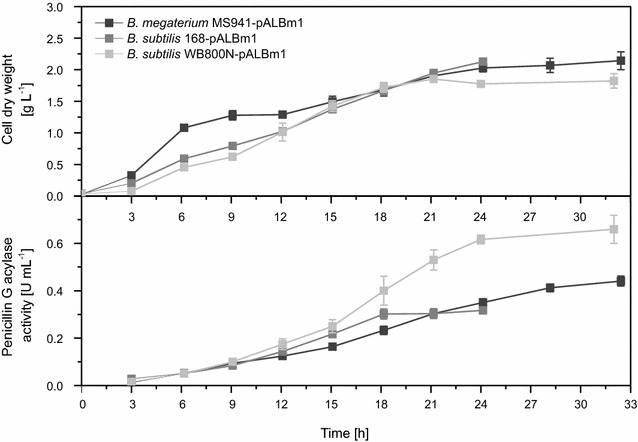
Time-dependency of cell dry weight and penicillin G acylase activity for cultivation of recombinant *Bacillus* strains. Cultivation took place in baffled shake flasks (150 mL culture volume) under aerobic conditions at 37 °C in LB medium containing 10 mg L^−1^ of tetracycline. Samples were taken at given time points to analyze cell dry weight [g L^−1^] and activity of penicillin G acylase [U mL^−1^] via enzymatic assay

Growth was similar for all investigated strains. All strains reached the stationary phase after 21 h with cell dry weights of about 2 g L^−1^ (Fig. 6). Production of active recombinant protein was demonstrated for all tested strains. Maximal extracellular Pac activities of 0.4 U mL^−1^ (24–32 h), 0.3 U mL^−1^ (18 h) and 0.65 U mL^−1^ (24–32 h) were detected for *B. megaterium* MS941, *B. subtilis* 168 and *B. subtilis* WB800N, respectively. Maximal Pac activities were observed after longer cultivation times compared to those required for maximal concentrations of D1.3 scFv. Here again *B.* *subtilis* 168 reached maximal product amount earlier compared to *B. megaterium* MS941 and *B. subtilis* WB800N (Fig. 6). The lowest Pac activities of 0.3 U mL^−1^ were observed with *B. subtilis* 168, which were even lower than with *B. megaterium* MS941 (0.4 U mL^−1^), probably showing the influence of highly active proteases, while the lack of eight proteases characterizing *B. subtilis* WB800N doubled the maximal activity to 0.65 U mL^−1^. The observed production and secretion behavior for Pac was similar for all tested strains compared to the antibody fragment production and secretion.

### Time-resolved comparison of recombinant D1.3 scFv secretion in different *B. subtilis* strains

Next, *B. subtilis* strains 168, DB431 and WB800N all transformed with pRBBm117 were individually cultivated in microtiter plates, shake flasks, and bioreactors (Table 1) investigating time-resolved growth behavior and secretion of D1.3 scFv. Recombinant *B. subtilis* 168 and *B. subtilis* DB431 grew faster with maximal specific growth rates of 0.42–0.53 h^−1^ in all three cultivation systems compared to recombinant *B. megaterium* and *B. licheniformis* (Additional file 1: Figure S3). Recombinant D1.3 scFv production and secretion was induced after 2.5–3.5 h, resulting in decreased specific growth rates of 0.16–0.27 h^−1^ until reaching 0.34–0.49 h^−1^ again about 4–6 h after induction. Stationary phase was already reached after 10–12 h with maximal cell dry weights of 1.5–2.0 g L^−1^. In contrast, growth of recombinant *B.* *subtilis* strain WB800N was slower (0.36–0.41 h^−1^). Also, induction of recombinant scFv secretion after 3.0–3.5 h of growth resulted in less decreased growth rate (0.22–0.29 h^−1^). Stationary phase was reached after 12–14 h, but lower maximal cell dry weights of 1.0–1.3 g L^−1^ were detected compared to the other two *B. subtilis* strains. During stationary phase, xylose was partly metabolized (1.0–2.5 g L^−1^) by all three *B. subtilis* strains after depletion of fructose (Additional file 1: Figures S4–S6), again indicating an efficient uptake of the sugar.

Cultivating *B. subtilis* strains in bioreactor, significant foam formation was fought by addition of overall 30 mg L^−1^ (168), 50 mg L^−1^ (DB431) and 100 mg L^−1^ (WB800N) of anti-foaming agent. The observed amount of foam can be explained by the high amount of extracellular proteins known to encourage foam formation [58] (Fig. 5b). The DO dropped to 0% 3 h (168) and 2 h (DB431) prior transition to stationary phase (Additional file 1: Figures S4, S5). Due to slower growth for WB800N, the DO of this strain only dropped down to 20% about 2 h before transition to stationary phase (Additional file 1: Figure S6) since a lower OTR into the cells was needed. The observed oxygen limitation in bioreactor cultivations resulted in the secretion of the fermentation product acetate in high amounts of 0.6 g L^−1^ (168), 0.7 g L^−1^ (DB431) and 0.9 g L^−1^ (WB800N) (Additional file1: Figures S4–S6). In contrast to that, culture volume reduction realized higher OTR and minimal DO values of 20–50% (168), 40% (DB431) and 80% (WB800N) in microtiter plates resulting in lower amounts of secreted acetate [0.1 g L^−1^ (168), 0.3–0.4 g L^−1^ (DB431), 0.7 g L^−1^ (WB800N)] compared to bioreactor cultivations. For all *B. subtilis* strains and cultivation systems, maximal acetate concentrations occurred simultaneously at transition to stationary phase. Finally, strains 168 and DB431 re-utilized secreted acetate completely in the stationary phase, resulting in another decline of DO in the bioreactors, but due to low concentrations of acetate this was not the case in microtiter plates (Additional file 1: Figures S4, S5). Strain WB800N only partly metabolized the acetate in the growth medium (Additional file 1: Figure S6). Secretion and re-utilization of acetate was described previously for another *B. subtilis* strain [59].

**Fig. 7:**
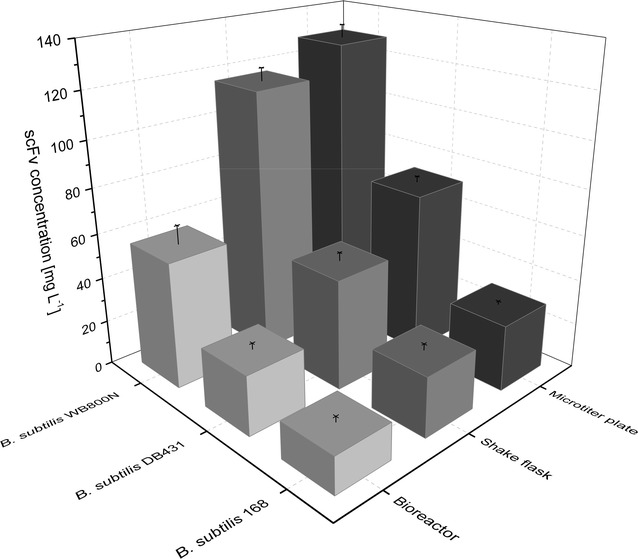
Maximal extracellular D1.3 scFv concentrations for recombinant *B. subtilis* 168, DB431 and WB800N. *B. subtilis* strains were transformed with plasmid pRBBm117 (P_*xylA*_^opt.^-*sp*_*lipA*_-*d1.3scFv*-*his*_*6*_). Cultivation took place in microtiter plates (1250 μL culture volume), baffled shake flasks (150 mL culture volume) and stirred tank bioreactors (2 L culture volume) under aerobic conditions at 37 °C. All cultivation systems were inoculated with cell concentrations (cell dry weight) of 0.0337 g L^−1^ in minimal medium containing 10 mg L^−1^ of tetracycline. Recombinant D1.3 scFv secretion was induced by the addition of 5 g L^−1^ of xylose at cell concentrations of around 0.10 g L^−1^. Extracellular concentration of D1.3 scFv [mg L^−1^] for *B. subtilis* 168, DB431 and WB800N was analyzed using ELISA. Further analyses are shown in Additional file 1: Figures S3–S6

**Fig. 8:**
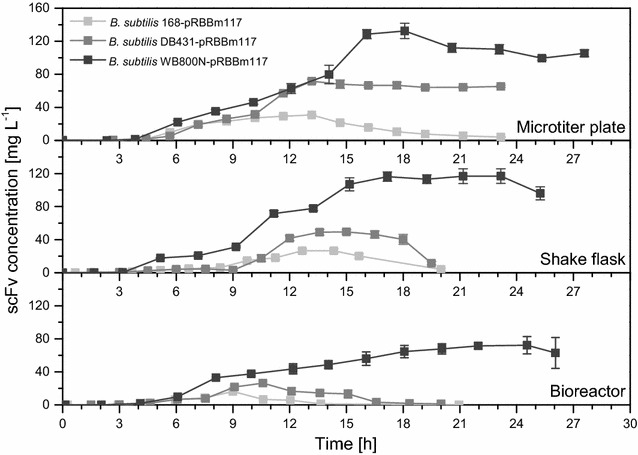
Time-dependent extracellular concentration of D1.3 scFv for the recombinant *B. subtilis* 168, DB431 and WB800N. Cultivation took place in microtiter plates (1250 µL culture volume), baffled shake flasks (150 mL of culture volume) and bioreactors (2 L culture volume) under aerobic conditions at 37 °C. All cultivation systems were inoculated with cell concentrations (cell dry weight) of 0.0337 g L^−1^ in minimal medium containing 10 mg L^−1^ of tetracycline. Recombinant D1.3 scFv secretion was induced by the addition of 5 g L^−1^ of xylose at cell concentrations of around 0.07 g L^−1^. Samples were taken at given time points to analyze extracellular concentration of D1.3 scFv [mg L^−1^] for *B. subtilis* 168, DB431 and WB800N using ELISA

Biologically active D1.3 scFv was secreted with all three recombinant *B. subtilis* strains in all three cultivation systems (Figs. 7, 8). Secretion was observed to be strongly (168) or mainly (DB431 and WB800N) growth-associated, since maximal concentrations were observed during or shortly after transition into stationary phase, respectively (Additional file 1: Table S1). Growth-associated secretion of a scFv with *B. subtilis* was described earlier with much lower product concentration although using a protease deficient strain [60]. In this study, maximal formed and secreted D1.3 scFv concentrations significantly differed depending on the used *B. subtilis* strain and the employed cultivation system. Maximal D1.3 scFv amounts in shake flasks were 30 mg L^−1^ (168, after 12–13 h), 50 mg L^−1^ (DB431, after 14 h) and 120 mg L^−1^ (WB800N, after 16–18 h), respectively, while for *B. subtilis* DB431 and WB800N, slightly higher concentrations were observed in microtiter plates (60 and 130 mg L^−1^) due to better OTRs. For bioreactors, lower maximal D1.3 scFv concentration of 20 mg L^−1^ (168), 30 mg L^−1^ (DB431) and 70 mg L^−1^ (WB800N) were obtained, which might be due to the limited supply of oxygen or a negative effect of the added anti-foaming agent on product formation or secretion [61]. Compared to *B. megaterium* MS941 and *B. licheniformis* MW3, higher D1.3 scFv concentrations were achieved in less cultivation time with all three *B. subtilis* strains.

*Bacillus subtilis* 168 did not only secrete the smallest amount of D1.3 scFv among the examined *B. subtilis* strains but its extracellular scFv concentration also strongly decreased during stationary phase for all cultivation systems. Especially in stirred tank bioreactors, scFv concentration dropped dramatically fast in stationary phase of cultivation, resulting in a quite narrow and small concentration profile. Here, degradation by proteases was strongly assumed, since *B. subtilis* 168 is known to produce and secrete high amounts of different proteases, especially at the end of growth phase [62]. Apparently, the absence of six proteases in *B. subtilis* DB431 resulted in higher and more constant scFv titer profiles, although degradation of scFv during stationary phase still occurred (Fig. 8). Finally, *B. subtilis* WB800N, lacking eight proteases, secreted the highest scFv amounts for all three cultivation systems and no significant degradation of scFv occurred in any cultivation system during stationary phase clearly indicating the positive influence of proteases deficiency on the recombinant protein production and secretion in this *B. subtilis* strain.

### Space–time-yield (STY) of D1.3 scFv secretion in *Bacillus*

**Fig. 9:**
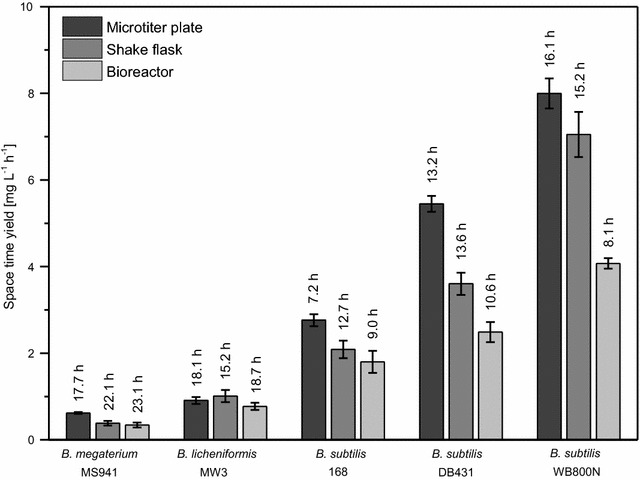
Maximal space–time-yield of extracellular D1.3 scFv. Space–time-yields (STY; [mg L^−1^ h^−1^]) were calculated as proportion of scFv concentration [mg L^−1^] and the corresponding cultivation time [h] for each examined *Bacillus* strain cultivated in microtiter plates, shake flasks and stirred tank bioreactors

For overall balancing and evaluation of the production and secretion of D1.3 scFv, the space–time-yield (STY) was calculated as the proportion of scFv concentration and corresponding cultivation time (Fig. 9). For recombinant *B. megaterium*, STY of about 0.35 mg L^−1^ h^−1^ for shake flasks and bioreactors and of 0.62 mg L^−1^ h^−1^ for microtiter plates were attained. With recombinant *B. licheniformis*, higher values of 1.01, 0.77 and 0.91 mg L^−1^ h^−1^ were observed. STY for recombinant *B. subtilis* were much higher. Here again, values increased from strain 168 over DB431 to WB800N. Cultivation in stirred tank bioreactors resulted in the lowest STY (1.8, 2.5 and 4.1 mg L^−1^ h^−1^) despite the short corresponding cultivation time. In microtiter plates, the highest values of 2.8, 5.4 and 8.0 mg L^−1^ h^−1^ were reached, even though these for shake flasks were only slightly lower (2.1, 3.6 and 7.0 mg L^−1^ h^−1^). The highest STY was reached with *B. subtilis* WB800N for all three cultivation systems. These STYs were about 15 times higher than that of the recombinant reference strain *B. megaterium* MS941.

## Discussion

In this study, different strains of the genus *Bacillus* were shown to efficiently produce and secrete the recombinant antibody fragment D1.3 scFv as well as penicillin G acylase Pac in three different cultivation systems. For this, all strains were successfully transformed with the identical plasmid encoding D1.3 scFv and Pac, respectively, together with an optimized *B. megaterium* xylose-inducible promoter system and *B. megaterium* originated signal peptides [63]. This clearly shows the broad interspecies application of an expression plasmid in *Bacillus*, which was not investigated so far.

Production and secretion of scFvs and scFabs with *B. megaterium* were examined before using its native xylose-inducible promoter system. With this system, 390 µg L^−1^ of anti-CRP scFv [64], 410 µg L^−1^ of D1.3 scFv [33] and 3.5 µg L^−1^ of the more complex D1.3 scFab were produced and secreted in their active forms by *B. megaterium* strain MS941 [64] deficient in the main extracellular protease NprM lacking around 98% of the overall extracellular protease activity [36]. When compared to the production and secretion by a *B. megaterium* strain additionally deficient in xylose metabolization [30], D1.3 scFv secretion was enhanced to 700 µg L^−1^ in shake flasks and even 3 mg L^−1^ in 3 L bioreactors [40, 65]. With *E. coli*, about 1–2 mg L^−1^ of D.13 scFv were recovered as soluble protein in the culture supernatant and as periplasmic fraction [66], showing the inefficient production in Gram-negative bacteria.

For *B. licheniformis*, no secretion of any scFv was described so far. For the here-used strain *B. licheniformis* MW3, scFv concentrations in the same range as for *B. megaterium* were achieved. Among the tested *B. subtilis* strains, highest amounts of active D1.3 scFv were secreted with the strain WB800N deficient in eight proteases. WB800N showed the highest product yield which was 8.7-fold higher than the best *B. megaterium* MS941 cultivation and 4.3- and 2.2-fold higher than for *B. subtilis* strains 168 and DB431, respectively. Deficiencies of *aprE*, *npr*, *epr*, *mpr*, *ispA* and *bpr* of *B. subtilis* DB431 lead to doubling of scFv concentration (up to 60 mg L^−1^) compared to *B. subtilis* 168 (up to 30 mg L^−1^). Additional deficiency in *nprB*, *vpr* and *wprA* in *B. subtilis* WB800N resulted in another doubling of scFv concentration (up to 130 mg L^−1^). This is the highest level of a recombinant antibody fragment for a Gram-positive bacterium described so far. In the literature, *B. subtilis* strains, mostly mutants of strain 168 with deletions of one or more protease genes, show very low overall extracellular protease activity leading to improved yields of various products compared to the wild type strain [21]. Secretion of up to 15 mg L^−1^ with *B. subtilis* was described before [53, 60, 67]. The three-protease-deficient *B. subtilis* WB30 was reported to secrete 3.5 mg L^−1^ and the six-protease-deficient WB600 5 mg L^−1^ of an scFv against digoxin [60]. With coproduction of intracellular and extracytoplasmic molecular chaperones, scFv titers of 12 mg L^−1^ were observed [67]. With *B. subtilis* WB700N, a seven-extracellular-protease-deficient derivate, secretion of scFv against fibrin even failed [53]. However, the combination of enhanced coproduction of molecular chaperones and additional inactivation of the cell-wall-associated protease WprA in the strain WB800HM[pEPP] resulted in scFv titers of up to 15 mg L^−1^ [53]. In addition, correlation of increased stability of the produced scFv with less secreted proteases was observed comparing *B. subtilis* 168, WB30 and WB600 [60]. In accordance with that, in this study higher and more constant scFv titer profiles were observed for the protease-deficient *B. subtilis* strains DB431 and WB800N.

Apart from *B. megaterium* and *B. subtilis*, production of extracellular recombinant antibody fragments has been reported only for *B. brevis* and *Lactobacillus* spp. With *B.* *brevis*, secretion of about 100 mg L^−1^ of a Fab against human urokinase-type plasminogen activator was reported [68]. *Lactobacillus* spp. were used to secrete 6 µg L^−1^ of Guy’s 13 scFv against a streptococcal adhesion molecule [69], 410 µg L^−1^ of codon-optimized antiviral 3D8 scFv [70] and 1 mg L^−1^ of an of llama heavy-chain (VHH) antibody fragment against rotavirus [71].

In contrast to *Bacillus*-based production systems mammalian cell cultures produce antibodies at g L^−1^ scale. However, much longer cultivation times of up to 3 weeks are needed. For example, using a human cell line 400–500 mg L^−1^ of IgG were produced within 10 days, corresponding to a STY of 1.66–2.08 mg L^−1^ h^−1^ [72]. IgG concentrations of 2.64 g L^−1^ (in 13 days) were realized with NSO cells, which corresponds to a STY of 8.46 mg L^−1^ h^−1^ [73]. For CHO cells up to 12.8 mg L^−1^ h^−1^ of IgG were described [74–76]. Since we observed maximal secretion with the tested *Bacillus* strains in only 9–21 h, the resulting STY of up to 8 mg L^−1^ h^−1^ are in the same order of magnitude as described for mammalian cells, showing the ability to compete with these cultivation systems for scFv-secretion. However, cultivation costs are characteristically smaller for the bacterial production systems.

Recombinant production of penicillin G acylase Pac from *B. megaterium* ATCC14945 in other *B. megaterium* [30] and *B. subtilis* [77–79] strains was shown previously, using various cultivation systems and mainly complex media. Here, we used the production of this enzyme for the confirmation of the results obtained for scFv-production and secretion. In agreement, the deficiency for eight proteases in *B. subtilis* WB800N led to a doubling of the maximal activity while the presence of all natural protease clearly reduced the amount of Pac in the medium, validating the assumptions made for D1.3 scFv production in protease-deficient *B. subtilis* strains. Overall, similar results were obtained in comparison to the recombinant secretion of D1.3 scFv, clearly confirming the general applicability of the introduced systems.

## Conclusions

Different tested strains of the genus *Bacillus* provided high potential microbial production systems to synthesize and export challenging heterologous proteins like antibody fragments and high molecular weight proteins at high titers in different cultivation systems: a microtiter plate approach (1250 μL culture volume), uncontrolled shake flasks (150 mL culture volume) and a controlled laboratory-scale stirred tank bioreactor (2 L culture volume). The employed plasmid originally optimized for the recombinant protein production in *B. megaterium* can also efficiently be used for *B. licheniformis* and *B. subtilis* strains, which demonstrates its interspecies application. Kinetics of D1.3 scFv production were successfully described with the Luedeking–Piret model for all tested *Bacillus* strains and cultivation systems. Highest production and secretion of the antibody fragment was attained in microtiter plates and shake flask because of their better oxygen transfer compared to the stirred tank bioreactor approach. *B. subtilis* WB800N, deficient in eight proteases, secreted 130 mg L^−1^ (STY of 8 mg L^−1^ h^−1^) of the recombinant antibody fragment D1.3 scFv, which is the highest concentration and STY of a recombinant antibody fragment for a Gram-positive bacterium described so far. Finally, interspecies application of the plasmid system were also shown for the secretion of a recombinant heterodimeric protein, the penicillin G acylase Pac.

## Methods

### Strains and plasmids

All strains and plasmids used in this study are listed in Table 2. For cloning procedures, *E. coli* strain DH10B acted as host (Life Technologies, Carlsbad, USA). *B. megaterium* strain MS941 is a derivate of the wild type strain DSM319 with a defined deletion in the gene encoding the major extracellular protease NprM [36, 80]. *B licheniformis* strain MW3, a derivate of the type strain DSM13, has defined deletions in genes encoding two type I restriction modification systems (HsdR1, HsdR2) to improve transformability and recombinant DNA stability [47]. *B. subtilis* 168, the most widely biotechnologically used *B. subtilis* strain, is originated from *B. subtilis* Marburg via X-ray-mutagenesis. It is tryptophan-requiring auxotroph [51]. *B. subtilis* strain DB431 is deficient in four extracellular and two intracellular proteases [52], while strain WB800N lacks six extracellular, one cell-wall-associated and one intracellular protease [53, 54]. For generation of the here used expression plasmid pRBBm117, the *D1.3scFv* gene fused to the coding sequences of the signal peptide of the *B. megaterium* lipase A [63] at its 5′-end, of a His_6_-tag at its 3′-end and of a terminator structure were liberated from plasmid pEJBmD1.3scFv [33] using the restriction enzymes *Bsr*GI and *Age*I. The fragment was cloned into similarly cut p3STOP1623hp [32] resulting in pRBBm117. For generation of expression plasmid pALBm1, the *pac* gene encoding the penicillin G acylase was amplified from genomic DNA of *B. megaterium* ATCC14945 using the primers pac_fw (5′-TACATATGTACAATGAAGACGAAGTGGCTAATATCA-3′) and pac_rv (5′-TATCAGAGCTCATCAATAGTATAGGCTC-3′). The *pac* gene including the native signal peptide was cloned into the shuttle vector p3STOP1623hp [32] using *Sac*I und *Bsr*GI.

**Table 2:** Strains and plasmids used in this study

Strain or plasmid	Description	Reference/source
*Escherichia coli* DH10B	F^−^ *endA1 deoR* ^+^ *recA1 galU galK16 nupG rpsL* Δ*(lac)X74* φ80*lacZΔM15 araD139* Δ*(ara, leu)7697 mcrA* Δ*(mrr*-*hsdRMS*-*mcrBC)* Str^R^ λ^−^	Invitrogen, Carlsbad, USA
*Bacillus megaterium* MS941	*Bacillus megaterium* DSM319 Δ*nprM*	[36]
*Bacillus megaterium* ATCC14945	Produces penicillin G acylase	American Type Culture Collection; Manassas, Virginia; USA
*Bacillus licheniformis* MW3	*Bacillus licheniformis* DSM13 (Δ*hsdR1*, Δ*hsdR2*)	[47]
*Bacillus subtilis* 168	*trpC2*	[51]
*Bacillus subtilis* DB431	*trpC2 nprE18 aprE epr bpr mpr ispA*	[52]
*Bacillus subtilis* WB800N	*nprE aprE epr bpr mpr::ble nprB::bsr Δvpr wprA::hyg cm::neo;* Neo^R^	[53, 54]
pEJBmD1.3scFv	Shuttle vector for the recombinant production and secretion of D1.3 scFv in *B. megaterium* (Tc^R^)	[33]
p3STOP1623hp	Shuttle vector for cloning in *E. coli* (Ap^R^) and production of target proteins in *B.* *megaterium* (Tc^R^) controlled by the optimized xylose-inducible promoter; P_*xylA*_^*opt*^-*mcs*-*stop; oriU/repU* (*B. cereus*)	[32]
pRBBm117	p3STOP1623hp-derivative: shuttle vector for the recombinant production and secretion of D1.3 scFv in *B.* *megaterium* (Tc^R^) controlled by the optimized xylose-inducible promoter; P_*xylA*_^*opt*^-*sp*_*lipA*_-*d1.3scFv*-*his*_*6*_-*stop, oriU/repU* (pBC16)	This work
pALBm1	p3STOP1623hp-derivative: shuttle vector for the recombinant production and secretion of penicillin G acylase in *B. megaterium* (Tc^R^) controlled by the optimized xylose-inducible promoter; P_*xylA*_^*opt*^-*sp*_*pac*_-*pac*-*stop, oriU/repU* (pBC16)	This work

### Isolation of genomic DNA from *B. megaterium* ATCC14945

For preparation of chromosomal DNA from *B. megaterium* ATCC14945, the JETquick Plasmid Miniprep Kit (Genomed, Löhne, Germany) was used according to the manufacturer’s instructions with some modifications. Cells from a cryogenic glycerol stock were grown overnight at 37 °C and 130 min^−1^ in 50 mL of LB medium in baffled flasks. Cells of 6 mL culture suspension were harvested and suspended in 250 µL TE(H) buffer (150 mM Tris–HCl, 20 mM EDTA, pH 8) before 20 µL of lysozyme solution (20 g L^−1^ lysozyme in TE(H) buffer) were added. Cells were incubated for 10 min at 37 °C and 750 min^−1^ before adding the next buffer. Before elution of DNA, the column was washed with 500 mL of GX buffer and 500 µL of G4 buffer.

### Transformation and strain maintenance

Unless mentioned here, molecular biology methods used in this study were described previously [81]. Plasmid DNA for the transformation of *B. megaterium* MS941, *B. licheniformis* MW3 and *B. subtilis* 168 was prepared from recombinant *E. coli* while plasmid DNA for transformation of *B. subtilis* strains DB431 and WB800N was prepared from *B. subtilis* 168 already carrying the plasmid pRBBm117. All DNA preparations were done by using the QIAgen Spin Miniprep Kit (QIAgen, Hilden, Germany) according to the manufacturer’s instructions with some modifications. For the preparation of plasmid DNA from Gram-positive *B.* *subtilis*, lysozyme (1 mg mL^−1^) was added to the resuspension buffer for suspending cells of 50 mL culture (OD_600_ of 1). Cells were then incubated for 10 min at 37 °C before adding the next buffer.

The protoplast transformation of *B. megaterium* was performed as outlined previously [82]. The protoplast transformation of *B. licheniformis* was performed according to Waschkau et al. [47]. All *B. subtilis* strains were transformed via induced natural competence. For this purpose, cells from a cryogenic glycerol stock were grown overnight at 37 °C and 230 min^−1^ in 10 mL competence medium in baffled flasks. The competence medium consists of 500 mL L^−1^ 2× SMM (Spizizen minimal medium), 5 g L^−1^ glucose, 100 mg L^−1^ tryptophan, 1.48 g L^−1^ MgSO_4_·7H_2_O, 200 mg L^−1^ casamino acids and 1.1 mg L^−1^ Fe-NH_4_-citrate. 2× SMM consists of 4 g L^−1^ (NH_4_)_2_SO_4_, 28 g L^−1^ K_2_HPO_4_, 12 g L^−1^ KH_2_PO_4_, 2 g L^−1^ Na-citrate·2H_2_O and 400 mg L^−1^ MgSO_4_·7H_2_O. 10 mL of fresh competence medium in a 100 mL baffled flask were inoculated with 600 µL of the overnight culture. Cells were grown for 3 h at 37 °C and 230 min^−1^ before 10 mL of pre-warmed starvation medium (500 mL L^−1^ 2× SMM, 5 g L^−1^ glucose, 1.48 g L^−1^ MgSO_4_·7H_2_O and 1.1 mg L^−1^ Fe-NH_4_-citrate) were added. After another 2 h of incubation under same conditions, 400 µL of the cell suspension were mixed with 1 µg of corresponding plasmid DNA and incubated for 1 h at 1000 min^−1^ and 37 °C. Finally, 100 µL were plated on LB agar with 10 mg L^−1^ of tetracycline and incubated overnight at 37 °C.

All strains were stored at −80 °C in a 30% (v/v) glycerol solution in cryogenic vials. For preparing cryogenic cultures, 50 mL of LB medium [81] with 10 mg L^−1^ of tetracycline for plasmid-carrying recombinant strains were inoculated with cell material from a LB agar plate and incubated at 37 °C and 230 min^−1^. During exponential phase (OD_600_ = 1–2), 32.5 mL of the cell suspension were mixed with 17.5 mL of glycerol [86% (v/v)], immediately frozen in 1 mL aliquots in liquid nitrogen and stored at −80 °C.

### Cultivation of recombinant strains

The minimal medium used in this study for production of recombinant D1.3 scFv contained 5 g L^−1^ d-fructose, 25 g L^−1^ (NH_4_)_2_SO_4_, 300 mg L^−1^ MgSO_4_·7H_2_O, 3.52 g L^−1^ KH_2_PO_4_, 5.3 g L^−1^ Na_2_HPO_4_, 10 mg L^−1^ of tetracycline and 2 mL L^−1^ trace element solution. For all *B. subtilis* strains, 16 mg L^−1^ tryptophan and 7.36 g L^−1^ glutamic acid were added. The trace element solution contained 424 mg MnCl_2_·4H_2_O, 678.4 mg CaCl_2_·4H_2_O, 16.8 mg FeSO_4_·4H_2_O, 1.4 mg CoCl_2_, 0.4 mg CuSO_4_·4H_2_O, 6.2 mg H_3_BO_3_, 1.25 mg ZnSO_4_·7H_2_O and 2.6 mg (NH_4_)_6_Mo_7_O_24_·4H_2_O per liter. For 100 mL of pre-cultures, an adequate amount of cryogenic culture was used as inoculum (100 µL for *B. megaterium*, 250 µL for *B. licheniformis*, 50 µL for *B. subtilis* 168 and DB431 and 150 µL for *B. subtilis* WB800N). These pre-cultures were grown in 500 mL baffled flasks at 37 °C and 130 min^−1^ for 18 h. Main cultures were inoculated with a pre-culture volume adjusting to an OD_600_ of 0.1. Inoculation culture was centrifuged (1500×*g*, 5 min, room temperature) before suspended in fresh minimal medium. Expression of the *D1.3* *scFv* was induced by addition of 5 g L^−1^ d-xylose at an OD_600_ of 0.3–0.4.

For shake flasks cultivation for production of recombinant D1.3 scFv, 150 mL of minimal medium were incubated in 500 mL baffled flasks (3 baffles) at 37 °C and 130 min^−1^ (50 mm orbital shaking diameter). Shake flask cultivations were carried out in triplicate. For bioreactor cultivation, 3 L bioreactors (Applikon, Schiedam, The Netherlands) with two six-bladed Rushton turbine impellers were used. Cultivation was performed with 2 L of minimal medium. Temperature (37 ± 0.1 °C), aeration rate (3.0 L min^−1^, 1.5 vvm), agitation speed (500 min^−1^) and pH value (pH 6.5 ± 0.1, adjusted with 2 M HCl and 2 M NaOH) were automatically kept constant. Dissolved oxygen (DO) was measured online. Bioreactor cultivations were carried out in duplicate (strains MS941, MW3, 168) and triplicate (strains DB431, WB800N), respectively. To prevent foam formation, anti-foaming agent (Ucolub^®^, FRAGOL GmbH & Co. KG, Mühlheim/Ruhr, Germany) was added when required. For microtiter plate cultivation, 1250 µL of minimal medium was transferred to each well of a 48 Well FlowerPlate^®^ (m2p-labs, Baesweiler, Germany) with optodes for pH and DO. Plates were covered with an adhesive gas-permeable membrane (Thermo Scientific, Dreieich, Germany) and incubated in a BioLector^®^ unit (m2p-labs, Baesweiler, Germany) at 37 °C and 1000 min^−1^ with an orbital shaking diameter of 3 mm. The calibration values for the measurement of pH and DO were obtained from calibration data sheets provided by m2p-labs. According to manufacturer (m2p-labs, Baesweiler, Germany), the oxygen transfer rate in 48 Well FlowerPlates^®^ comes to 35–40 mmol L^−1^ h^−1^ for the used conditions (see above).

Samples of cultivation supernatant were taken for HPLC analysis (fructose, xylose and acetate) as well as for quantitative ELISA test (D1.3 scFv) as described below. For this, cell suspension (1–2 mL) was centrifuged at 9000×*g*, 5 min, 4 °C. Sterile filtered (polyvinylidene fluoride, 0.2 μm pore size, Roth, Karlsruhe, Germany) supernatant was stored at −20 °C.

Cultivation of all plasmid strains for the secretion of recombinant penicillin G acylase was performed in shake flasks as described for the production of D1.3 scFv above but took place in LB medium [81]. Main cultivations were performed in duplicates. Samples of cultivation supernatant were taken for penicillin G acylase activity assay as described below. For this, 1 mL cell suspension was centrifuged (9000×*g*, 5 min, 4 °C), supernatant was filtered (polyvinylidene fluoride, 0.2 μm pore size, Roth, Karlsruhe, Germany) and stored at 4 °C before use.

### OD_600_ and cell dry weight

Cell concentration was determined as optical density at a wavelength of 600 nm using a Libra S11 Visible Spectrophotometer (Biochrom GmbH, Berlin, Germany). Cell dry weight (CDW) was measured via gravimetric analysis. Here, 20 mL culture volume (duplicate) were pelleted (1500×*g*, 15 min, 4 °C) in Corex glass tubes, washed twice with distilled water to remove salts and dried at 80 °C for 48 h. The relationship between CDW and OD_600_ was determined as CDW [g L^−1^] = *a* [g L^−1^] · OD_600_ [–] with coefficient *a* equals 0.223 ± 0.007 g L^−1^ (*B. megaterium* MS941), 0.40 ± 0.01 g L^−1^ (*B. licheniformis* MW3) and 0.337 ± 0.002 g L^−1^ (all *B. subtilis* strains used in this study), respectively.

### High performance liquid chromatography (HPLC) analysis

Sugars (xylose, fructose) were quantified by an HPLC system (HitachiElite LaChrom, Krefeld, Germany) equipped with a Metacarb 87 °C column (Varian, Palo Alto, CA, USA) as stationary phase and Millipore H_2_O as mobile phase at 0.6 mL min^−1^ and 85 °C with a retention time of approximately 11.7 min (xylose) and 13.4 min (fructose). Detection was performed using a refractive index detector (RI) detector. Acetate were also measured using an HPLC system (Hitachi Elite LaChrom, Krefeld, Germany) equipped with an Aminex HPX 87 H column (Biorad, Hercules, CA, USA) as the stationary phase and 12.5 mM H_2_SO_4_ as mobile phase at 0.5 mL min^−1^ and 45 °C with a retention time of approximately 18.5 min. Detection was performed using a RI detector.

### Enzyme-linked immunosorbent assay (ELISA)

ELISA was used to quantify recombinant D1.3 scFv in the culture supernatant. Nunc MaxiSorp^®^ flat-bottom 96 well plates (Nunc, Wiesbaden, Germany) were coated with 100 µL of 10 mg L^−1^ hen egg white lysozyme in PBS buffer (145 mM NaCl, 7.5 mM Na_2_HPO_4_·2H_2_O and 2.2 mM NaH_2_PO_4_·2H_2_O) per well overnight at 4 °C. Coated wells were washed three times with 100 µL of PBS-T 0.05% [0.05% (v/v) Tween 20 in PBS] and blocked with 2 g L^−1^ skim milk powder in PBS-T 0.1% [0.1% (v/v) Tween 20 in PBS] for 1.5 h, followed by three times washing with 100 µL PBS-T 0.05%. Samples of 100 µL were incubated for 1.5 h, followed by three times washing with 100 µL of PBS-T 0.05%. Detection was performed with 100 µL of monoclonal Mouse anti penta His HRP conjugate (0.1 ng µL^−1^ in PBS-T 0.1%) and visualized with 100 μL of tetramethylbenzidine (TMB) substrate solution. Substrate solution consisted of 10 parts of TMB-A solution (3 mM H_2_O_2_ and 200 mM potassium citrate at pH 4) and 0.5 parts of TMB-B solution (41 mM 3,3′,5,5′-tetramethylbenzidine in DMSO). The staining reaction was stopped by adding 100 μL of 1 M H_2_SO_4_. The absorbance at 450 nm and scattered light at 620 nm were measured using a microtiter plate reader (SUNRISE, Tecan, Crailsheim, Germany). The absorbance at 620 nm was subtracted.

On each plate, a calibration standard of purified D1.3 scFv was applied. Standards were purified from culture supernatant with immobilized metal affinity chromatography (IMAC) and quantified by densiotometric analysis as described below. Samples for ELISA were measured in adequate dilutions (with PBS) and quantified according to the standard via five-parameter logistic analysis.

### Penicillin G acylase activity assay

100 µL of substrate solution (0.6 g L^−1^ of synthetic substrate 2-nitro-5-[(phenylacetyl)amino]-benzoic acid, 9.41 mM NaH_2_PO_4_, 40.59 mM Na_2_HPO_4_, pH 7.5) were added into a QS ultra-micro cuvette (10 mm light pass; Hellma Analytics, Müllheim, Germany) in a tempered (37 °C) cuvette holder of an UV–Vis spectrometer (Jasco V-650, Jasco, Groß-Umstadt, Germany). Addition of 11.11 µL of pre-warmed (37 °C) cell-free supernatant started the enzymatic reaction to 2-nitro-5-aminobenzoic acid, absorbing at 405 nm (extinction coefficient ε of 8.98 cm^2^ µmol^−1^). Change of absorption dA/dt [min^−1^] at 405 nm was measured every second for 1 min and was used to calculate enzymatic activity EA [µmol cm^−3^ min^−1^]

$${\text{EA = }}\frac{{\frac{\text{dA}}{\text{dt}} \cdot {\text{V}}_{\text{R}} }}{{{\varepsilon} \cdot {\text{d }} \cdot {\text{V}}_{\text{E}} }};$$

where, V_R_ is the reaction volume [cm^3^], V_E_ the sample volume [cm^3^] and d equals the light pass [mm]. Enzyme activity was expressed in U mL^−1^ with one Unit U equals the enzyme amount to convert 1 µmol of 2-nitro-5-[(phenylacetyl)amino]-benzoic acid per minute at 37 °C.

### Immobilized metal affinity chromatography (IMAC), sodium dodecyl sulfate polyacrylamide gel electrophoresis (SDS-PAGE) and densiotometric analysis

Recombinant D1.3 scFv was purified via IMAC. For this purpose a gravity flow column (BIORAD, Hercules, California, USA) was loaded with 1 mL Chelating Sepharose Fast Flow (Amersham Biosciences, Freiburg, Germany), washed with 10 mL H_2_O, incubated with 2 mL 100 mM NiSO_4_ for 10 min and washed with 5 mL H_2_O followed by 5 mL washing buffer (50 mM NaCl, 20 mM Tris–HCl pH 8.6). The nickel charged Sepharose was transferred to 50–100 mL of culture supernatant containing 7 mM of imidazole and mixed for 90 min at 4 °C. The Sepharose was pelleted by centrifugation (1500×*g*, 5 min, 4 °C) and washed with 3 mL washing buffer. Elution was performed with 3 × 1 mL elution buffer (50 mM EDTA in washing buffer). 18 µL of each fractions were analyzed by SDS-PAGE analysis (12% of acrylamide) as described before [83]. Gels were stained with SimplyBlue™ SafeStain according to the manufacturer’s instructions for the microwave procedure (Thermo Fisher Scientific Inc., Waltham, Massachusetts, USA). Band visible on the stained SDS PAGE corresponding to recombinant D1.3 scFv in the elution fractions from IMAC were quantified by densitometric analysis (ImageJ 1.48v, National Institute of Health, Bethesda, USA) related to a BSA standard (10–100 mg L^−1^).

### Precipitation of extracellular proteins

Extracellular proteins of *B. megaterium* and *B. subtilis* strains cultivated in shake flasks were precipitated by incubating 1.5 mL culture supernatant with 660 mg (NH_4_)_2_SO_4_ for 2 h at 4 °C in an overhead shaker. After centrifugation (9000×*g*, 30 min, 4 °C), precipitated proteins were suspended in 80 µL of 8 M urea supplemented with 27 µL of 4-times SDS loading buffer. 15 µL of this were analyzed by SDS-PAGE as described above.

### Determination of the volumetric mass transfer coefficient (*k*_*L*_*a*) in bioreactors

*k*_*L*_ *a* value in bioreactors was determined with sulfite oxidation method, which based on oxidation of Na_2_SO_3_ to Na_2_SO_4_ by oxygen. Measurement was performed under cultivation conditions (described above) without cells. 40 mg L^−1^ of Co(NO_3_)_2_ were added as catalyst. DO was monitored online with an oxygen sensor (Visiferm DO 225, Hamilton, Höchst im Odenwald, Deutschland). Calibration to 100% was performed under oxygen saturation condition (DO_max_ = 6.784 mg L^−1^ at 37 °C). Complete oxygen depletion was achieved by addition of 10 mL of a 200 g L^−1^ Na_2_SO_3_ stock solution [84]. As soon as the oxidation was completed the DO increased again. Plotting ln(DO_max_ − DO) against time, the linear slope was used to determine the *k*_*L*_ *a* value.

### Kinetics analysis of D1.3 scFv production

The Luedeking–Piret model was used for modeling the D1.3 scFv production and secretion [85]. The observed experimental product profile from cultivation start to the time point of maximal D1.3 scFv concentration was approximated by the numerical integration of the Luedeking–Piret equation

$${\text{P}}_{\text{n}} = {\text{P}}_{{{\text{n}}{ - }1}} + \left( {\upalpha \cdot \frac{{{\text{X}}_{\text{n}} { - } {\text{X}}_{{{\text{n}}{ - }1}} }}{{{\text{t}}_{\text{n}} { - } {\text{t}}_{{{\text{n}}{ - }1}} }} + \upbeta \cdot {\text{X}}_{\text{n}} } \right)\left( {{\text{t}}_{\text{n}} { - } {\text{t}}_{{{\text{n}}{ - }1}} } \right);$$

here, t_n_ and t_n − 1_ are the present and previous time steps of integration. P_n_ and P_n − 1_ are concentrations of the product D1.3 scFv and X_n_ and X_n − 1_ the cell dry weights at the present and previous time steps. The growth-associated Luedeking–Piret constant α [${\text{mg}}_{\text{scFv}} \,{\text{g}}_{{_{\text{cells}} }}^{ - 1}$] equals the biomass-related product yield coefficient Y_P/X_ for D1.3 scFv formation. The non-growth-associated Luedeking–Piret constant β [${\text{mg}}_{\text{scFv}} \,{\text{g}}_{{_{\text{cells}} }}^{ - 1} \,{\text{h}}^{{{ - }1}}$] represents the specific production rate for specific growth rate µ = 0 h^−1^. These biological reaction parameters were set so that the coefficient of determination R^2^ was maximal, representing the goodness of fit of the model concentrations of D1.3 scFv compared to the experimental concentrations. Calculation was performed with the Generalized Reduced Gradient algorithm of the add-in program Solver for Microsoft Excel 2010 (Microsoft, Redmund, WA, USA).

## Additional file

**Additional file 1.** Additional tables and figures.

## Abbreviations

α (≡Y_P/X_): growth-associated Luedeking–Piret constant and biomass related product yield coefficient [${\text{mg}}_{\text{scFv}} \,{\text{g}}_{{_{\text{cells}} }}^{ - 1}$]
β: non-growth-associated specific production rate for specific growth rate µ = 0 [${\text{mg}}_{\text{scFv}} \,{\text{g}}_{{_{\text{cells}} }}^{ - 1} \,{\text{h}}^{{{ - }1}}$]
*B. megaterium*: *Bacillus megaterium* (for *B. licheniformis* and *B. subtilis* as well)
CDW: cell dry weight [g L^−1^]
DO: dissolved oxygen [%]
ELISA: enzyme-linked immunosorbent assay
HPLC: high-performance liquid chromatography
*k*_*L*_*a*: volumetric oxygen transfer coefficient [h^−1^]
µ: specific growth rate [h^−1^]
OTR: oxygen transfer rate [mg L^−1^ h^−1^]
P: concentration of the product D1.3 scFv
scFv: single-chain fragment variable
SDS-PAGE: sodium dodecyl sulfate polyacrylamide gel electrophoresis
STY: space–time-yield [mg_scFv_ L^−1^ h^−1^]
t: time [h]
X: cell concentration (cell dry weight) [g L^−1^]
Y_P/X_: biomass related product yield coefficient [mg_scFv_ g_cells_^−1^]
