# Supplementary material for: Recombinant production of the antibody fragment D1.3 scFv with different Bacillus strains
Source: Microb Cell Fact. 2017 Jan 23;16:14. doi: 10.1186/s12934-017-0625-9 (PMC5259949; doi:10.1186/s12934-017-0625-9)
Supplement: Supplementary file 1 — Additional file 1. Additional tables and figures. [file 12934_2017_625_MOESM1_ESM.docx]

**Additional file 1**

**Recombinant production of the antibody fragment D1.3 scFv with different *Bacillus*strains**

Antonia Lakowitz, Rainer Krull, Rebekka Biedendieck

**Table S1: Luedeking-Piret model for D1.3 scFv production with different *Bacillus* in microtiter plates, shake flasks and bioreactors.** While α [mg_scFv_ g_cells_^‑1^] is the growth-associated constant for D1.3 scFv formation, β [mg_scFv_ g_cells_^‑1^ h^‑1^] represents the non-growth-associated specific production rate. The coefficient of determination R^2^ indicates the goodness of fit of the model concentrations of D1.3 scFv compared to the experimental concentrations

| Production strain | Cultivation system | α [mg g^‑1^] | β [mg g^‑1^ h^‑1^] | R^2^ [-] |
| --- | --- | --- | --- | --- |
| *B. megaterium* MS941 | Microtiter plate  Shake flask  Bioreactor | 0.28  0.13  0.00 | 0.79  0.41  0.31 | 0.911  0.971  0.890 |
| *B. licheniformis* MW3 | Microtiter plate  Shake flask  Bioreactor | 0.00  3.29  0.00 | 1.19  1.29  0.51 | 0.949  0.940  0.724 |
| *B. subtilis* 168 | Microtiter plate  Shake flask  Bioreactor | 22.66  16.23  17.65 | 0.00  0.00  0.00 | 0.734  0.988  0.967 |
| *B. subtilis* DB431 | Microtiter plate  Shake flask  Bioreactor | 6.210  4.209  6.900 | 4.083  2.809  1.974 | 0.968  0.898  0.974 |
| *B. subtilis* WB800N | Microtiter plate  Shake flask  Bioreactor | 6.72  37.49  29.12 | 11.54  6.17  2.49 | 0.964  0.978  0.948 |

**Figure S1: Time-dependency of various parameters for cultivation of recombinant *B. megaterium* MS941.** Cultivation took place in microtiter plates (1,250 µL culture volume), baffled shake flasks (150 mL culture volume) and bioreactors (2 L culture volume) under aerobic conditions at 37 °C in minimal medium containing 10 mg L^‑1^ of tetracycline. Samples were taken at given time points to analyze concentration [g L^‑1^] of fructose, xylose and acetate using HPLC. Dissolved oxygen was measured online in microtiter plates and bioreactors. For microtiter plates, dissolved oxygen was averaged over all filled wells at the corresponding time point. For bioreactors, dissolved oxygen of one exemplary reactor is presented, since averaging over all bioreactors is not rational due to individual addition of anti-foaming agent.

**Figure S2: Time-dependency of various parameters for cultivation of recombinant *B. licheniformis* MW3.** Cultivation took place in microtiter plates (1,250 µL of culture volume), baffled shake flasks (150 mL culture volume) and bioreactors (2 L culture volume) under aerobic conditions at 37 °C in minimal medium containing 10 mg L^‑1^ of tetracycline. Samples were taken at given time points to analyze concentration [g L^‑1^] of acetate, xylose and fructose concentration using HPLC. Dissolved oxygen was measured online in microtiter plates and bioreactors. For microtiter plates, dissolved oxygen was averaged over all filled wells at the corresponding time point. For bioreactors, dissolved oxygen of one exemplary reactor is presented, since averaging over all bioreactors is not rational due to individual addition of anti-foaming agent.

**S3: Time-dependency of cell dry weight for the recombinant *B. subtilis* 168, DB431 and WB800N.** Cultivation took place in baffled shake flasks (150 mL culture volume), bioreactors (2 L culture volume) and microtiter plates (1,250 µL culture volume) under aerobic conditions at 37 °C in minimal medium containing 10 mg L^‑1^ of tetracycline. All cultivation systems were inoculated with cell concentrations (cell dry weight) of 0.0337 g L^‑1^ in minimal medium containing 10 mg L^‑1^ of tetracycline. Recombinant D1.3 scFv secretion was induced by the addition of 5 g L^‑1^ of xylose at cell concentrations of around 0.07 g L^‑1^. Samples were taken at given time points to analyze cell dry weight [g L^‑1^] for *B. subtilis* 168, DB431 and WB800N.

**S4: Time-dependency of various parameters for cultivation of recombinant *B. subtilis* 168.** Cultivation took place in microtiter plates (1,250 µL culture volume), baffled shake flasks (150 mL culture volume) and bioreactors (2 L culture volume) under aerobic conditions at 37 °C in minimal medium containing 10 mg L^‑1^ of tetracycline. Samples were taken at given time points to analyze concentration [g L^‑1^] of acetate, xylose and fructose concentration using HPLC. Dissolved oxygen was measured online in microtiter plates and bioreactors. For microtiter plates, dissolved oxygen was averaged over all filled wells at the corresponding time point. For bioreactors, dissolved oxygen of one exemplary reactor is presented, since averaging over all bioreactors is not rational due to individual addition of anti-foaming agent.

**S5: Time-dependency of various parameters for cultivation of recombinant *B. subtilis* DB431.** Cultivation took place in microtiter plates (1,250 µL culture volume), baffled shake flasks (150 mL culture volume) and bioreactors (2 L culture volume) under aerobic conditions at 37 °C in minimal medium containing 10 mg L^‑1^ of tetracycline. Samples were taken at given time points to analyze concentration [g L^‑1^] of acetate, xylose and fructose concentration using HPLC. Dissolved oxygen was measured online in microtiter plates and bioreactors. For microtiter plates, dissolved oxygen was averaged over all filled wells at the corresponding time point. For bioreactors, dissolved oxygen of one exemplary reactor is presented, since averaging over all bioreactors is not rational due to individual addition of anti-foaming agent.

**S6: Time-dependency of various parameters for cultivation of recombinant *B. subtilis* WB800N.** Cultivation took place in microtiter plates (1,250 µL culture volume), baffled shake flasks (150 mL culture volume) and bioreactors (2 L culture volume) under aerobic conditions at 37 °C in minimal medium containing 10 mg L^‑1^ of tetracycline. Samples were taken at given time points to analyze concentration [g L^‑1^] of acetate, xylose and fructose concentration using HPLC. Dissolved oxygen was measured online in microtiter plates and bioreactors. For microtiter plates, dissolved oxygen was averaged over all filled wells at the corresponding time point. For bioreactors, dissolved oxygen of one exemplary reactor is presented, since averaging over all bioreactors is not rational due to individual addition of anti-foaming agent.
